# A strategy for scaling up access to comprehensive care in adults with Chagas disease in endemic countries: The Bolivian Chagas Platform

**DOI:** 10.1371/journal.pntd.0005770

**Published:** 2017-08-18

**Authors:** Maria-Jesus Pinazo, Jimy Pinto, Lourdes Ortiz, Jareth Sánchez, Wilson García, Ruth Saravia, Mirko-R Cortez, Silvia Moriana, Enric Grau, Daniel Lozano, Joaquim Gascon, Faustino Torrico

**Affiliations:** 1 International Health Department, ISGlobal, Barcelona Center for International Health Research, (CRESIB), Hospital Clínic-Universitat de Barcelona, Spain; 2 Fundación CEADES, Cochabamba, Bolivia; 3 Chagas Disease Global Coalition, Barcelona, Spain; Instituto de Ciências Biológicas, Universidade Federal de Minas Gerais, BRAZIL

## Abstract

**Background:**

Bolivia has the highest prevalence of Chagas disease (CD) in the world (6.1%), with more than 607,186 people with *Trypanosoma cruzi* infection, most of them adults. In Bolivia CD has been declared a national priority. In 2009, the Chagas National Program (ChNP) had neither a protocol nor a clear directive for diagnosis and treatment of adults. Although programs had been implemented for congenital transmission and for acute cases, adults remained uncovered. Moreover, health professionals were not aware of treatment recommendations aimed at this population, and research on CD was limited; it was difficult to increase awareness of the disease, understand the challenges it presented, and adapt strategies to cope with it. Simultaneously, migratory flows that led Bolivian patients with CD to Spain and other European countries forced medical staff to look for solutions to an emerging problem.

**Intervention:**

In this context, thanks to a Spanish international cooperation collaboration, the Bolivian platform for the comprehensive care of adults with CD was created in 2009. Based on the establishment of a vertical care system under the umbrella of ChNP general guidelines, six centres specialized in CD management were established in different epidemiological contexts. A common database, standardized clinical forms, a and a protocolized attention to adults patients, together with training activities for health professionals were essential for the model success. With the collaboration and knowledge transfer activities between endemic and non-endemic countries, the platform aims to provide care, train health professionals, and create the basis for a future expansion to the National Health System of a proven model of care for adults with CD.

**Results:**

From 2010 to 2015, a total of 26,227 patients were attended by the Platform, 69% (18,316) were diagnosed with *T*. *cruzi*, 8,567 initiated anti-parasitic treatment, more than 1,616 health professionals were trained, and more than ten research projects developed. The project helped to increase the number of adults with CD diagnosed and treated, produce evidence-based clinical practice guidelines, and bring about changes in policy that will increase access to comprehensive care among adults with CD. The ChNP is now studying the Platform’s health care model to adapt and implement it nationwide.

**Conclusions:**

This strategy provides a solution to unmet demands in the care of patients with CD, improving access to diagnosis and treatment. Further scaling up of diagnosis and treatment will be based on the expansion of the model of care to the NHS structures. Its sustainability will be ensured as it will build on existing local resources in Bolivia. Still human trained resources are scarce and the high staff turnover in Bolivia is a limitation of the model. Nevertheless, in a preliminary two-years-experience of scaling up this model, this limitations have been locally solved together with the health local authorities.

## Author summary

Bolivia has the highest prevalence of Chagas disease (CD) in the world (6.1%), with more than 607,186 people with *Trypanosoma cruzi* infection. In Bolivia, the management of CD has been declared a national priority. In 2009, the Chagas National Program (ChNP) had neither a protocol nor a clear directive for diagnosis and treatment of adults. The Chagas Platform has been built as a model for comprehensive care of adults with CD. From 2010 to 2015, a total of 26,227 patients were attended by the Platform, 69% (18,316) were diagnosed with *T*. *cruzi*, 8,567 initiated anti-parasitic treatment, more than 1,616 health professionals were trained. More than ten research projects were developed. The project has also produced evidence-based clinical practice guidelines, and brings about changes in policy that will increase access to comprehensive care among adults with CD. The ChNP is now studying the Platform’s health care model to adapt and implement it nationwide. It is an experience of collaboration and knowledge transfer between endemic and non-endemic countries.

## Introduction

Chagas disease (CD) is caused by the parasite *Trypanosoma cruzi* and is one of 17 recognized neglected tropical diseases. Updated findings published in 2015 [1] estimate that between 6 and 7 million people worldwide have *T*. *cruzi* infection and that 25 million remain at risk of infection. Originally limited to Latin America, CD is now a global health problem as a result of migration flows from traditional endemic zones [2]. Bolivia has the highest prevalence in the world (6.1%), and the disease is endemic in 60% of the country [1]. The latest published data show that 607,186 persons in Bolivia are estimated to have CD and that a further 586,434 people are at risk, the number of new cases is estimated around 8,700 every year (8,087 for vector-borne transmission and 616 newborns with *T*.*cruzi* via congenital transmission).[1] Between 2 to 3% of those infected people develop cardiac and/or digestive complications every year. [3] Annually the number of estimated deaths caused by CD is around 388.[4] It is estimated that CD is responsible for 13% of deaths of people between 15 and 75 years. [3] However, the real magnitude of the problem remains unknown. Even the reduction in domiciliary infestation by the vector to below 3% in most parts of the country [5], it does not necessary correlate with advances in patient care. With rates below 3% of infestation we would have expected greater advances in the number of treated people, as treatment of positive patients is recommended by the ChNP when infestation rates are below this threshold. However the number of treated patients is very low and CD still remains an important unsolved public health problem.

The two drugs available for the aetiological treatment of CD are benznidazole and nifurtimox. While both are highly efficacious in newborns and children [6], there are concerns about their efficacy in adults [7], even if the results of recent studies indicate that efficacy is higher than previously reported [8] and that treatment decreases congenital transmission of *T*. *cruzi*.[9]

Considering that the lack of early treatment has important implications not only for individual patients, but also in terms of public health, implementation of a model to increase the number of adult patients diagnosed and treated and provide comprehensive care was prioritized [10,11]. Therefore, in 2009, a collaborative initiative was implemented by the Barcelona Centre for International Health Research (ISGlobal) and the Fundación Ciencia y Estudios Aplicados para el Desarrollo en Salud y Medio Ambiente (CEADES). This initiative was known as the Bolivian Chagas Platform.

The objective of this manuscript is to present the Chagas Platform as a model for comprehensive care of adults with CD and to show how its implementation can increase the number of people who have access to diagnosis and treatment. Healthcare coverage data are provided in order to quantify the impact of the initiative, and the limitations and lessons learned from this experience are described.

### Main gaps in provision of health care for patients with CD in 2009

**Fig 1:**
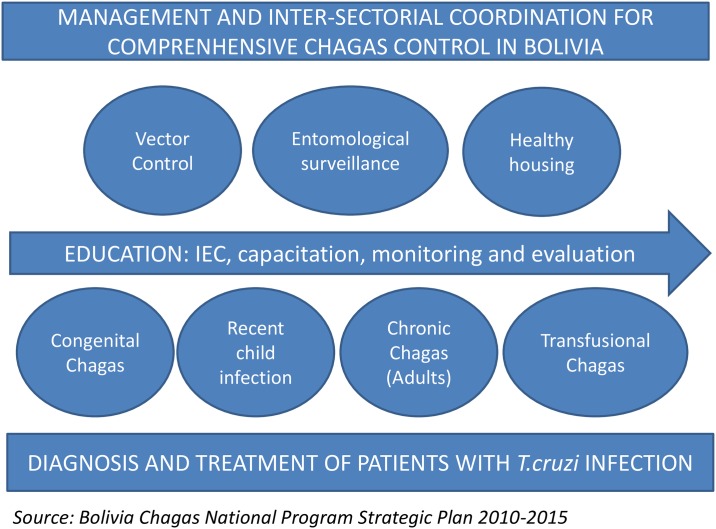
Comprehensive overview of the national Chagas program.

The prevention and control of CD in Bolivia was declared a national priority in Law 3374 dated March 23, 2006 [12]. However, no additional regulations were developed. The Chagas National Programme (ChNP), the responsible body for the prevention, diagnosis and treatment of CD in the country, elaborated drafted its Strategic Plan 2010–2015 [13]. Fig 1 provides a holistic and inter-sectorial overview of the Strategic Plan.

Given the epidemiological situation of Bolivia in the 1990s (infestation rates >50%), the priority area was vector control, which was financed by the Inter-American Development Bank (Banco Interamericano de Desarrollo, BID) from 1999 to 2006. This support included treatment of children but not adults with chronic CD, and the control of congenital disease (supported with funds from the Belgian Government until 2009) was prioritized.[14] Diagnosis and treatment of children under 18 years old should in theory be provided by primary healthcare centers in Bolivia, however many centers do not systematically provide this service. Even though CD affected mainly adults in Bolivia, no model for care of adults with chronic infection was defined, and therefore primary health center do not contemplate etiological treatment for this population.

In 2009, the ChNP reported that 178,012 persons had been screened. Most were pregnant women who were monitored for congenital transmission, although only 3,103 were treated (10% of those confirmed as having *T*. *cruzi* infection in the same year, representing only around 0.5% of all estimated patients with the infection) (Table 1).

**Table 1:** Diagnosis and treatment of CD in Bolivia in 2009*.

Target Population	Number of persons screened	Number of positive cases for *T*. *cruzi* infection	Number of persons treated
**Pregnant Women**	106,714	23,421	-
**Under <1 year**	15,073	303	207
**> 1 year < 5 years**	11,563	457	144
**>5 years < 15 years**	22,307	1,762	1,097
**>15 years**	22,355	4,938	1,655
**Total**	**178.012**	**30.881**	**3.103**

*Data from Bolivian Chagas National Program 2013.[15]

Additionally, health professionals’ knowledge of CD was limited. Since their training included information on the very frequent adverse effects of aetiological treatment and the autoimmune origin of cardiac involvement [16], medical staffs were reluctant to recommend aetiological treatment of chronic CD in adults. Consequently, treatment of adults was neglected, as occurred in other endemic countries. Today, there is sufficient evidence on the role of *T*. *cruzi* in triggering and sustaining the inflammatory response [8, 16] and, therefore, on the importance of early anti-parasitic treatment.

Furthermore, the lack of information on the benefits of treatment, together with fear and an alternative understanding of CD by at-risk persons, limited patients’ active demand for treatment [17,18]. Additionally, access to healthcare was hampered by the absence of symptoms, the non-specific nature of symptoms when present, and limited access to health centres, especially in rural settings.

### The platform: A strategy for increasing access to diagnosis and treatment of Chagas disease

Following a change in international consensus, **the Chagas Platform** was developed as a joint initiative that arose from the need to offer diagnosis and anti-parasitic treatment to adult patients in the chronic phase of *T*. *cruzi* infection.[19,20] In 2009, ISGlobal (Barcelona, Spain) and CEADES (Cochabamba, Bolivia) pooled their expertise in the comprehensive management of adult patients by developing a care model with the Bolivian Ministry of Health and Bolivian state universities to collaborate in research and training of health professionals.

The primary objective of the Chagas Platform is to contribute to the control of CD, and the model designed to achieve this objective is based on 4 pillars:

1. provision of health care
2. creation of expertise in management of CD and building the capacity for research
3. training health professionals in the management of CD
4. promotion of educational activities in the community

The Chagas Platform is therefore considered a translational model in which provision of care is the initial trigger of research needs, thus initiating a circular cycle where the results of research are applied in to healthcare and are used to train staff and effect changes in health policy.

In this manuscript, we focus on comprehensive care and staff training as critical components for future scaling up of access to diagnosis and treatment.

Comprehensive healthcare based on agreed protocols was initially provided in vertical, dedicated structures for adults. Even when these structures were located in existing health structures, they were conceived as specific units for CD, instead of as units for integrating care of CD patients in the normal outpatient care circuit. This strategy made it possible to create centres of expertise and ensured sufficient capacity to increase the number of people diagnosed and treated. It simultaneously generated a critical mass of patients that allowing to pilot the use of comprehensive care strategies that could subsequently be integrated in the national health system and to advance in key research areas. Additionally, the results of the Program revealed the magnitude of the problem and the need for a national strategy for patients in the chronic phase of CD.

There are currently six centres in three highly endemic departments of Bolivia: Tarija (one), Chuquisaca (one), and Cochabamba (four: two in rural areas and two in urban or semi-urban areas). The centres were established with different organizational set-ups that varied depending on the local partners that committed to the project in each area. Although each set-up has its particularities, they all share the same protocols and database. The network of Chagas Platform centres in both rural and urban areas enables patients to be transferred from one geographical area to another. These circuits ensure better coverage and improved access to healthcare.

The Chagas Platform centres offer their services free of charge. The project was built in collaboration with the Spanish Agency for International Development Cooperation (AECID) and contributions from local partners. The ChNP covers drug costs. Of the 35 persons working in the six centres, two are covered by SEDES (the departmental health authority in Chuquisaca) and eight by the Juan Misael Saracho University (Tarija); the remaining 25 are covered by ISGlobal and CEADES with AECID funds. Local authorities are expected to progressively assume the cost of human resources in the future.

**Fig 2:**
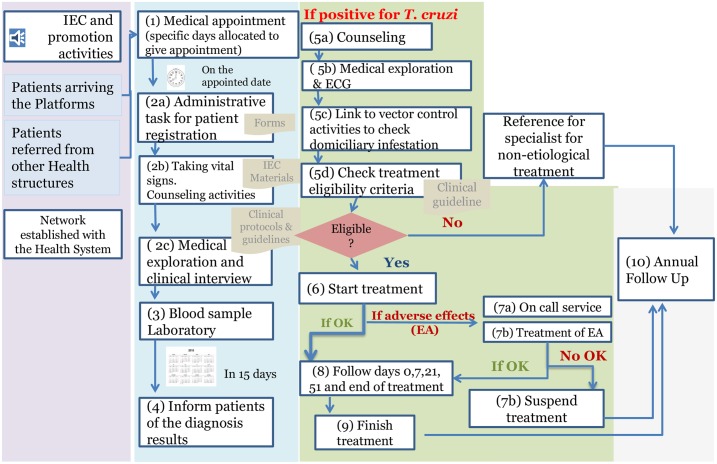
Healthcare roadmap for a patient entering the Chagas Platform.

The success of the model relies on the protocols and clinical guidelines used (see Fig 2).

The main elements are as follows:

- A comprehensive approach that includes counseling, Information, Education, and Communication (IEC) activities, and vector control monitoring, which is managed in coordination with departmental health authorities (SEDES) as key elements in diagnosis and treatment.
- Clinical evaluation before treatment, including ECG and physical examination to establish disease stage and determine whether the patient fulfils aetiological treatment criteria. Strict eligibility criteria to reduce risk during treatment (e.g., dietary indications and avoiding holidays). During aetiological treatment, the activities that have shown positive results for ensuring adherence and controlling potential adverse effects [21, 22] are as follows: medical evaluation on days 0, 7, 21, and 51 and at the end of treatment; an on-call system to manage adverse events in the early stages; and close follow-up (phone and messaging tools to ensure appointments are kept).
- A referral system to cover patients who need non-aetiological treatment owing to cardiac or gastrointestinal involvement.
- The possibility of follow-up with the same criteria under similar protocols in different parts of Bolivia and Barcelona (Spain).
- Annual follow-up to control potential progression of damage.

Another key element for the success of the program is the capacity of the staff to provide quality healthcare for CD patients. As health professionals were not sufficiently well trained in CD during their formal education, training of staff on current protocols became critical. After the first centres acquired expertise, the Chagas Platform started offering primary care staff a 1-week training program in the centres to learn the protocols.

Besides, the training and implementation of operational research included in the model has a relevant role giving to the National Health System personnel elements to analyze and reformulate priorities in Public Health interventions.

**Fig 3:**
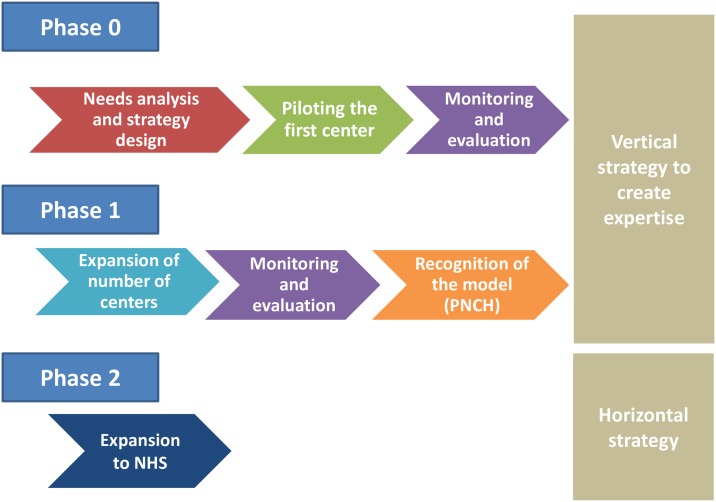
Stages of development of the intervention.

Finally, as the pilot project proved effective and acceptable, the Chagas Platform healthcare model has been expanded to primary healthcare centres since 2015, and a new strategy based on the network of centres managing CD was established, with the intention of expanding coverage in diagnosis and treatment in remote areas. The stages of development of the intervention have been reflected in Fig 3.

In addition to the adaptation of the protocols for managing CD in the health system care centres, this new horizontal approach was based on the training of health professionals (physicians, nurses, and biochemists) in these protocols and on referrals and counter-referral circuits between primary and specialized care centres.

## Results

Results from 2010 to 2015. Since the implementation of the Chagas Platform in 2009, a total of 26,227 adult patients have been attended in Bolivia, in the Platform centres. Around 69% had *T*. *cruzi* infection (18,316). To date, 8,567 patients have started treatment and, on average, 80% have received the complete course. Data regarding coverage of patients with *T*. *cruzi* infection are summarized in Table 2.

**Table 2:** Care provided from 2010 to 2015.

Data	2010	2011	2012	2013	2014	2015	TOTAL
**New patients attended**	864	3,868	5,062	5,746	5,222	5,465	26,227
Patients with confirmed *T*. *cruzi* infection(%)*	600(69.4%)	2,328(60.2%)	3,843(75.9%)	4,749(82.6%)	3,069(58.7%)	3,727(68.1%)	18,316(69.8%)
Patients who started aetiological treatment(%)**	385(64%)	807(34.6%)	1,123(29.2%)	2,339(49.2%)	1,868(60.8%)	2,045(54.8%)	8,567(32.7%)
**Number of visits**	3,967	8,443	13,729	20,390	20,286	21,230	88,045
**Personnel who underwent the 1-week training program**	10	40	57	61	55	80	303
**Personnel trained in management of CD**	10	40	199	296	210	155	910

*% of Patients attended;

**% of positive patients who start treatment

The number of patients was initially low because only two centres were functioning at the outset. The number of centres increased gradually until 2013, when the sixth and latest centre was opened. The increased demand has put strain on the system (organization, logistics, regulation of stocks, appointments) and the appropriate amount of medication has not always been available. Additionally, a benznidazole stock shortage in 2012 accounted for the low number of persons who started treatment during that year. Apart from the poor availability of drugs, which still limits the number of people who can be treated, the annual gap between persons with a positive diagnosis and persons treated can also be explained by the non-fulfilment of eligibility criteria and less importantly patient’s reluctance to be treated.

Strict fulfilment of inclusion criteria has improved adherence to treatment. On average, 80% of persons who initiated treatment finished the 60-day course, and conscientious follow-up ensured that data on adherence were excellent. Around 10% of patients left treatment voluntarily and around 10% of patients were advised to stop treatment owing to adverse drug reactions (ADRs) that were partially controlled with symptomatic treatment. Benznidazol and nifurtimox ADRs have been pointed out as one of the main problems for adherence to treatment, and due to the relevance of the topic, the description of them in the context of the Platform model will be described in deep in a separate manuscript.

The demand for the Platform was directly related to the implementation of community information activities. Since 2010, more than 25,000 people have received direct information about CD while in the healthcare process at Platform centres. Additionally, more than 3,500 people attended community information sessions. More than the half of these sessions was in the original language (mainly Quechua).

The percentage of people attending the centres that had *T*. *cruzi* infection was higher than expected, and most of those who were treated with benznidazole had excellent adherence to treatment.

The latest available data from the ChNP reveal the limitations of the ChNP for covering existing demand: in 2014, 29,052 adults were diagnosed with *T*. *cruzi* infection in Bolivia, and only 4,444 were treated (S1). Most of these patients (1,868, 42%) were treated in the Chagas Platform. [23]

In the areas where the Chagas Platform has its centres, yearly screening ranges from 0.7% to 2.1% of the estimated number of *T*.*cruzi* infected people. Almost two-thirds of current adult treatment is provided in the Platform centres, thus making them an important support structure for the ChNP in providing adult diagnosis and treatment.

Despite the quantitative and qualitative improvement in CD healthcare, the annual number of treated patients is less than 0.5% of the estimated total requiring treatment. There is also a considerable gap between people with *T*. *cruzi* infection and the number of people treated (only 10% among all the people with *T*. *cruzi* infection diagnosis). In this sense, the benefits of the project lie in the fact that it comprises a defined healthcare package, with concrete protocols to manage adults in the chronic phase of CD, that has proven effective and can be expanded to the National Health System.

Unfortunately, the total number of patients treated in Bolivia increased by only 14% between 2009 and 2015. While a favourable trend was observed for adults, the number of newborns and children treated decreased [15] Expanding the model to the primary health care system could reverse this trend. In collaboration with the Bolivian Ministry of Health, this is the next step proposed by CEADES and ISGlobal, with the support of AECID.

Since 2010, more than 1,600 health professionals have been trained in the specific management of patients with CD. A training fellowship program has been implemented between Universidad Mayor de San Simon (Bolivia) and Barcelona University, and five international professional exchanges have taken place since 2011. Specialized conferences and specific training activities were held during a conference in 2014, and more than 500 people participated.

## Discussion

### Lessons learned and future perspectives

Even if the Chagas Platform has proven to be highly effective, Platform centres have limited human resources to cover current demand in the management of CD in Bolivia. As recommended by the WHO, the main strategy for increasing access to healthcare requires the diagnosis and treatment of CD to be incorporated into the national health system, as part of their regular activities. In Bolivia, the recent establishment of a network including primary healthcare centres in rural areas following easy and realistic protocols is already showing positive results: it enables the management of CD in adults to be standardized and access to healthcare for people living in remote areas to be improved. To date, the integral CD healthcare model used in the Platform centres has been accepted for adaptation by the national health system before being implemented nationwide. Current Platform activities such as prevention, diagnosis, treatment, IEC, and training have been included in the implementation of the proposed model in national primary health centres. The main lessons learned from the implementation are as follows:

- Collaboration and coordination between primary care networks, the Chagas program, and other relevant local partners such as universities and local government is a key issue, not only for implementation, but also for the sustainability of the model. In this regard, intersectoral collaboration with vectorial control strategies (house refurbishment, disinsection, …) and education system have an important role in the comprehensive manage of this disease from a sociological point of view.
- The program should be introduced initially in places where strategic alliances can be established. The care model has the benefit of being able to be adapted to various scenarios; while this has brought clear benefits, it has also made coordination between the parties involved more complex.
- It is advisable to ensure supply of the necessary drugs before launching a diagnosis and treatment campaign. Centres should be able to provide additional testing on-site rather than referring patients elsewhere (e.g., laboratory tests, ECG).
- A system to manage referrals and counter-referrals should be established.
- Extending the network of centres offering CD services is crucial if access to care is to be scaled up.
- This type of project should have a long-term perspective, as it implies changes not only in systems, but also in the beliefs, attitudes, and behaviours of medical staff and patients. The major investment necessary at the beginning requires political engagement and ensured future sustainability through integration in the system.
- Social participation is key for the success of any intervention of this nature. At this point, to increase the silence demand of the civil society by IEC strategies contribute to better control CD as a Public Health problem.

The increase in the number of people diagnosed and treated must be made carefully in order to avoid excessive strain on the health system, which has to adapt gradually to growing demand. Finally, as CD is a neglected disease, several external factors can hamper patient management. The poor availability of current drugs (benznidazole and nifurtimox) and the lack of new and better-tolerated medicines are key limiting factors. Furthermore, the lack of biomarkers of response to treatment hinders research on new drugs [24]. Fortunately, in the last five years, international public and private initiatives have been launched to develop new drugs and to implement clinical trials, as have studies focused on biomarkers of cure and/or response to treatment, some of which have been performed in the Chagas Platform.

### Concluding remarks

Despite not being the only valid strategy, the Chagas Platform has proven to be a model of care for patients with *T*. *cruzi* infection that has been adopted by the Bolivian government. Moreover, the Chagas Platform has brought the benefits of reinforcing research capacity and training health professionals. Linking care, training, and research at the operational level is a very powerful tool that keeps health professionals updated and motivated. Additionally, the comprehensiveness of the program in the healthcare system should prove useful in other health-related issues.

The expertise that has formed the basis of guidelines and protocols is now being expanded to the national health system, thus highlighting the success of the program and enabling diagnosis and treatment to be scaled up.
